# Supplementary figures and images for: Exosomes from uterine fluid promote capacitation of human sperm
Source: PeerJ. 2024 Apr 25;12:e16875. doi: 10.7717/peerj.16875 (PMC11056104; doi:10.7717/peerj.16875)

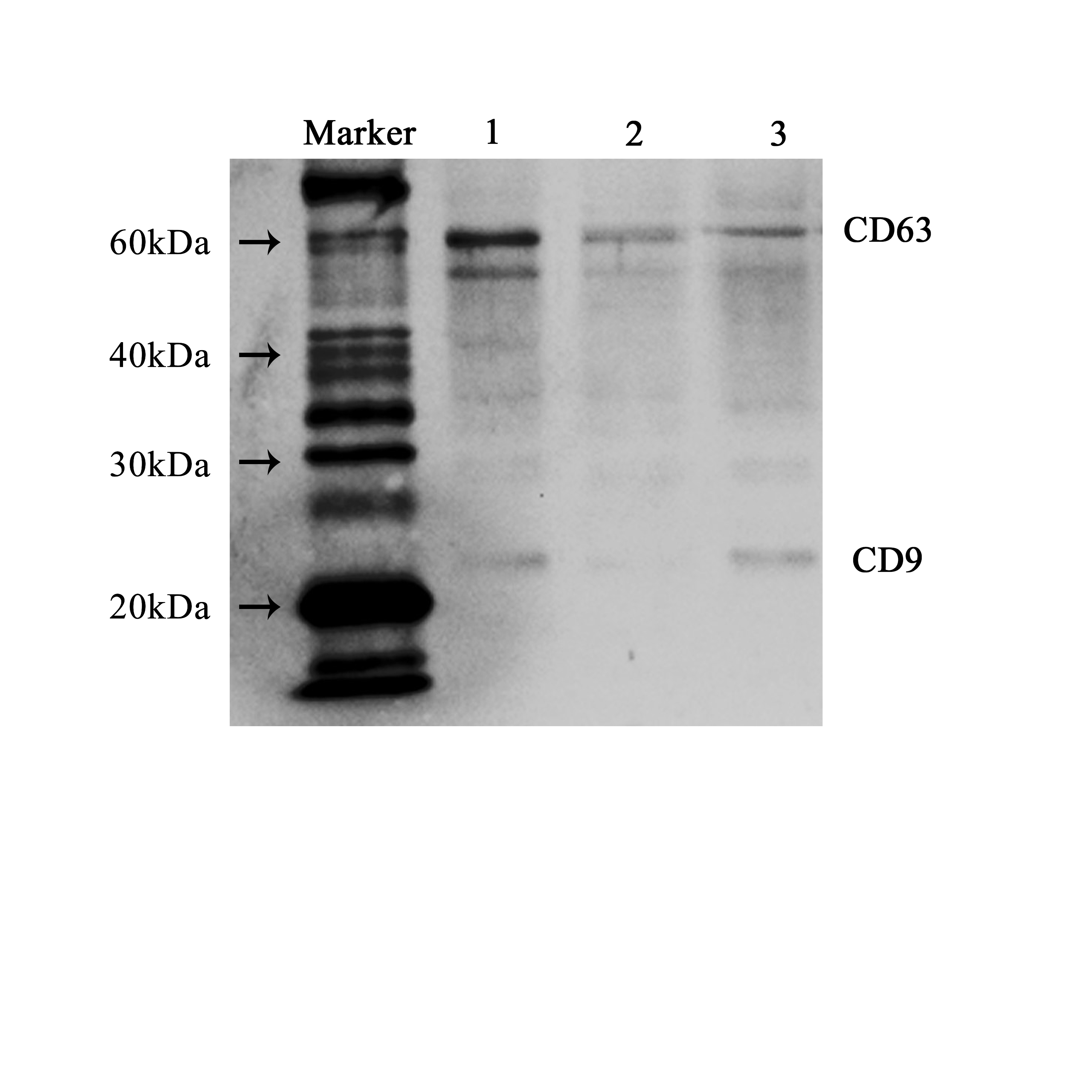

Supplement: Supplemental Information 4 [file peerj-12-16875-s004.jpg]
